# Prevalence of symptoms in 1512 COVID-19 patients: have dizziness and vertigo been underestimated thus far?

**DOI:** 10.1007/s11739-022-02930-0

**Published:** 2022-01-30

**Authors:** Mirko Aldè, Stefania Barozzi, Federica Di Berardino, Gianvincenzo Zuccotti, Dario Consonni, Umberto Ambrosetti, Marina Socci, Simona Bertoli, Alberto Battezzati, Andrea Foppiani, Diego Zanetti, Lorenzo Pignataro, Giovanna Cantarella

**Affiliations:** 1 Department of Clinical Sciences and Community Health, University of Milan, Milan, Italy; 2 Audiology Unit, Department of Specialist Surgical Sciences, Fondazione IRCCS Ca’ Granda Ospedale Maggiore Policlinico, Via Pace 9, 20122 Milan, Italy; 3 Department of Pediatrics, Children’s Hospital “Vittore Buzzi”, Azienda Socio Sanitaria Territoriale (ASST) Fatebenefratelli, Milan, Italy; 4 “L. Sacco” Department of Biomedical and Clinical Sciences, University of Milan, Milan, Italy; 5 Epidemiology Unit, Fondazione IRCCS Ca’ Granda Ospedale Maggiore Policlinico, Milan, Italy; 6 Department of Food Environmental and Nutritional Sciences (DeFENS), International Center for the Assessment of Nutritional Status (ICANS), University of Milan, Milan, Italy; 7 Obesity Unit and Laboratory of Nutrition and Obesity Research, Department of Endocrine and Metabolic Diseases , IRCCS (Scientific Institute for Research, Hospitalization, and Healthcare) Italian Auxologic Institute (IAI), Milan, Italy; 8 Otolaryngology Unit, Department of Specialist Surgical Sciences, Fondazione IRCCS Ca’ Granda Ospedale Maggiore Policlinico, Milan, Italy

**Keywords:** Dizziness, Vertigo, COVID-19, SARS-CoV-2, Presyncope, Balance Disorders

## Abstract

The relationship between SARS-CoV-2 infection and dizziness is still unclear. The aim of this study is to assess the prevalence and characteristics of dizziness and vertigo among patients with mild-to-moderate COVID-19. Patients discharged from the emergency rooms with a confirmed SARS-CoV-2 diagnosis were assisted by daily telephone calls until nasopharyngeal swab negativization, and specific symptoms concerning balance disorders were investigated through targeted questions posed by experienced physicians. The study included 1512 subjects (765 females, 747 males), with a median age of 51 ± 18.4 years. New-onset dizziness was reported by 251 (16.6%) patients, among whom 110 (43.8%) complained of lightheadedness, 70 (27.9%) of disequilibrium, 41 (16.3%) of presyncope, and 30 (12%) of vertigo. This study analyzed in detail the prevalence and pathophysiological mechanisms of the different types of balance disorders in a large sample, and the results suggest that dizziness should be included among the main symptoms of COVID-19 because one-sixth of patients reported this symptom, with females being significantly more affected than males (20.3 vs 12.9%, *P* < 0.001). Most cases of dizziness were attributable to lightheadedness, which was probably exacerbated by psychophysical stress following acute infection and mandatory quarantine. Vertigo should not be underestimated because it might underlie serious vestibular disorders, and disequilibrium in elderly individuals should be monitored due to the possible risk of falls.

## Introduction

Coronavirus disease 19 (COVID-19), which was first reported by officials in Wuhan City (China) in December of 2019, has quickly spread to all continents, thus causing a global pandemic with more than 5 million deaths worldwide since then [1].

Women and men of all ages are susceptible to SARS-CoV-2 (severe acute respiratory syndrome coronavirus 2) infection, but the elderly population with comorbidities has a significantly higher risk of serious complications [2]. The symptoms of SARS-CoV-2 infection are nonspecific and can range from very mild to severe, with a broad spectrum of clinical manifestations [2–5]. In most affected patients, the infection is paucisymptomatic; however, in some cases, it can progress to pneumonia, acute respiratory distress syndrome, and multiorgan dysfunction [2].

While some neurological symptoms, such as anosmia and dysgeusia, have been widely correlated with COVID-19 [3], the association with dizziness still appears unclear.

In April 2020, Mao et al. were the first authors to describe dizziness as the most common neurological symptom of COVID-19 based on reports for 36 of 214 hospitalized patients (16.8%) [6]. In a subsequent study by Viola et al., who implemented online questionnaires, 34 of 185 patients (18.4%) reported balance disorders after being diagnosed with COVID-19 [7]. Sia et al. also suggested that dizziness with unsteadiness while walking might be the only early clinical manifestation of SARS-CoV-2 infection [8].

In a systematic review, which included a total of 14 studies and 141 patients, Saniasiaya et al. found that dizziness had a prevalence ranging from 4 to 30%, although in most patients, balance disorders were not investigated and analyzed thoroughly [9]. A meta-analysis on nine papers, published by Jafari et al., demonstrated that the occurrence rate of dizziness in COVID-19 patients was 12.2% [10]. Another review of the literature showed that most of the studies were of poor quality and based on case reports or included retrospective surveys that referred to self-report questionnaires and patient recall [11]. Furthermore, the terms dizziness and vertigo were often incorrectly used interchangeably, thus leading to confusion about the actual prevalence and preventing the accurate identification of the origin, vestibular or otherwise, of balance disorders [11].

Despite the mentioned reports, neither the *World Health Organization* (WHO) [4] nor the *Centers for Disease Control and Prevention* (CDC) [5] have included dizziness in the list of symptoms suggestive of COVID-19.

The aim of this study is to investigate in detail the prevalence and pathophysiological mechanisms of vertigo and other nonvestibular causes of dizziness, which are traditionally classified as disequilibrium, presyncope, and lightheadedness [12], among consecutive patients with mild-to-moderate SARS-CoV-2 infection diagnosed in the Metropolitan City of Milan.

## Materials and methods

The present study included consecutive SARS-CoV-2-positive patients evaluated from October 1, 2020, to March 31, 2021, by “*The Operations Center for Discharged Patients*” (in Italy known as “COD19”, the acronym of “Centrale operativa dimessi COVID-19”).

COD19 is a virtual hospital model that provides assistance to patients through a dedicated medical telephone monitoring center, thus providing active home surveillance following discharge from the main hospital wards and emergency rooms of the Metropolitan City of Milan. This service was provided through a telephone exchange that is active 12 h a day, 7 days a week. The patients discharged with a confirmed diagnosis of SARS-CoV-2 infection were contacted by physicians daily via telephone to (1) ensure constant monitoring of several parameters and symptoms; (2) promptly recognize the most critical clinical or social conditions; and (3) provide psychological support.

Each patient was assisted from the day of diagnosis until the end of the quarantine period, which for Lombardy corresponded to the negativization of the nasopharyngeal swab or to the 21st day after the first positive nasopharyngeal swab (in the absence of symptoms for at least the last week). Information on age, sex, body mass index (BMI: kg/m^2^) and smoking status was carefully collected. The medical history of the patients was also obtained from the hospital and emergency department discharge letters.

This study included all patients with:

- Age ≥ 18 years;
- SARS-CoV-2 infection, confirmed by nasopharyngeal swabs processed by reverse transcription polymerase chain reaction (RT-PCR);
- mild-to-moderate severity of COVID-19.

Mild severity was defined by the absence of clinical or radiological evidence of pneumonia [13].

Moderate severity was characterized by clinical or radiological evidence of viral pneumonia but with respiratory rate and blood oxygen saturation still within the normal range of < 30 breaths/min and ≥ 94%, respectively [13].

The study excluded patients with:

- severe (blood oxygen saturation < 94%, respiratory rate > 30 breaths/min) to critical disease, who required hospitalization;
- a recent history (< 1 year) of dizziness;
- symptoms most likely due to previous or concomitant pathologies, such as a recent history of neoplasia or neurological or cardiovascular disease, not directly attributable to SARS-CoV-2 infection; and
- symptoms clearly caused by medications, head trauma, or surgical interventions.

Patients in compliance with the regulations of Lombardy but who decided to conclude mandatory quarantine 21 days after the diagnosis (being asymptomatic for at least the last week) without performing a control nasopharyngeal swab were also excluded from the study.

The symptoms investigated during home surveillance and analyzed in the present study were anorexia, anosmia, arthralgia, asthenia, cough, diarrhea, dysgeusia, dyspnea, fever, headache, myalgia, nausea, ocular symptoms, pharyngodynia, psychiatric symptoms, rhinitis, and dizziness.

- We considered the following definitions:
- Fever: axillary temperature ≥ 37.5 °C (99.5 °F).
- Ocular symptoms: presence of at least one of photophobia, epiphora, burning eyes, foreign body sensation, and blurred vision.
- Psychiatric symptoms: the presence of at least one of anxiety, depression, irritability, a lack of motivation, impaired concentration, mood changes, and psychoses.

According to the quality-of-symptoms approach [12], dizziness, a generic term used by patients to describe an altered sense of relationship with space, was distinguished into *vertigo*,* disequilibrium*, *presyncope*, and *lightheadedness*, which are defined as follows [12, 14–16]:

- Vertigo: a symptom of vestibular asymmetry that the patient experiences as an illusion of spinning motion of the surrounding environment (“objective vertigo”) or self-motion (“subjective vertigo”).
- Disequilibrium: a sense of imbalance, often described as feeling unsteady or wobbly, which occurs mostly when walking.
- Presyncope: a sudden onset symptom of near-fainting lasting for seconds to minutes, which is reported as “nearly blacking out” or “passing out without actual loss of consciousness”.
- Lightheadedness: a nonspecific dizziness, described as “feeling woozy and giddy, or disconnected from the environment”.

Patients with alarming symptoms, such as vomiting, diplopia, dysarthria, visual or hearing loss, were referred by medical phone operators to the nearest emergency room for the necessary investigations.

Listening carefully to the patients' descriptions of their symptoms and gathering additional information (onset, duration, trigger factors) from specific questions allowed physicians accustomed or professionally trained to manage balance disorders to formulate a hypothesis about the subtype of dizziness.

In this study, dizziness and its subcategories were also analyzed in more detail to evaluate the (1) clinical characteristics and prevalence based on sex, age group (18–39 years, 40–59 years, ≥ 60 years), smoking and BMI and (2) possible associations with the other general symptoms due to COVID-19.

The present study was conducted according to the World Medical Association’s Declaration of Helsinki and approved by the ethical committee of the University of Milan. Written informed consent was obtained from all participants.

### Statistical analysis

We used the chi-squared test to analyze categorical variables. Statistical analyses were performed with Stata 16 software (StataCorp. 2019). A *P* value of < 0.05 was accepted as statistically significant.

## Results

A total of 1512 patients (765 females, 747 males) met the inclusion criteria, and they had a median age of 51 (± 18.4) years (range 18–100). The mean period of active home surveillance was 24.8 (± 4.6) days.

The characteristics of the study population are summarized in Table 1.

**Table 1:** Characteristics of the study population

Variable	Patients, *N* (%)
Sex	
Female	765 (50.6)
Male	747 (49.4)
Age (years)	
< 40	417 (27.6)
40–59	595 (39.4)
≥ 60	500 (33.1)
Smoking	
Never	1011 (66.9)
Former	363 (24.0)
Current	138 (9.1)
BMI (kg/m^2^)	
< 18.5	27 (1.8)
18.5–24.9	740 (48.9)
25.0–29.9	643 (42.5)
≥ 30	102 (6.7)

No sex differences were found among the age groups (*P* = 0.98). Males were more frequently former or current smokers (*P* < 0.001) and had a higher BMI (*P* = *0.006*) than females.

**Fig. 1:**
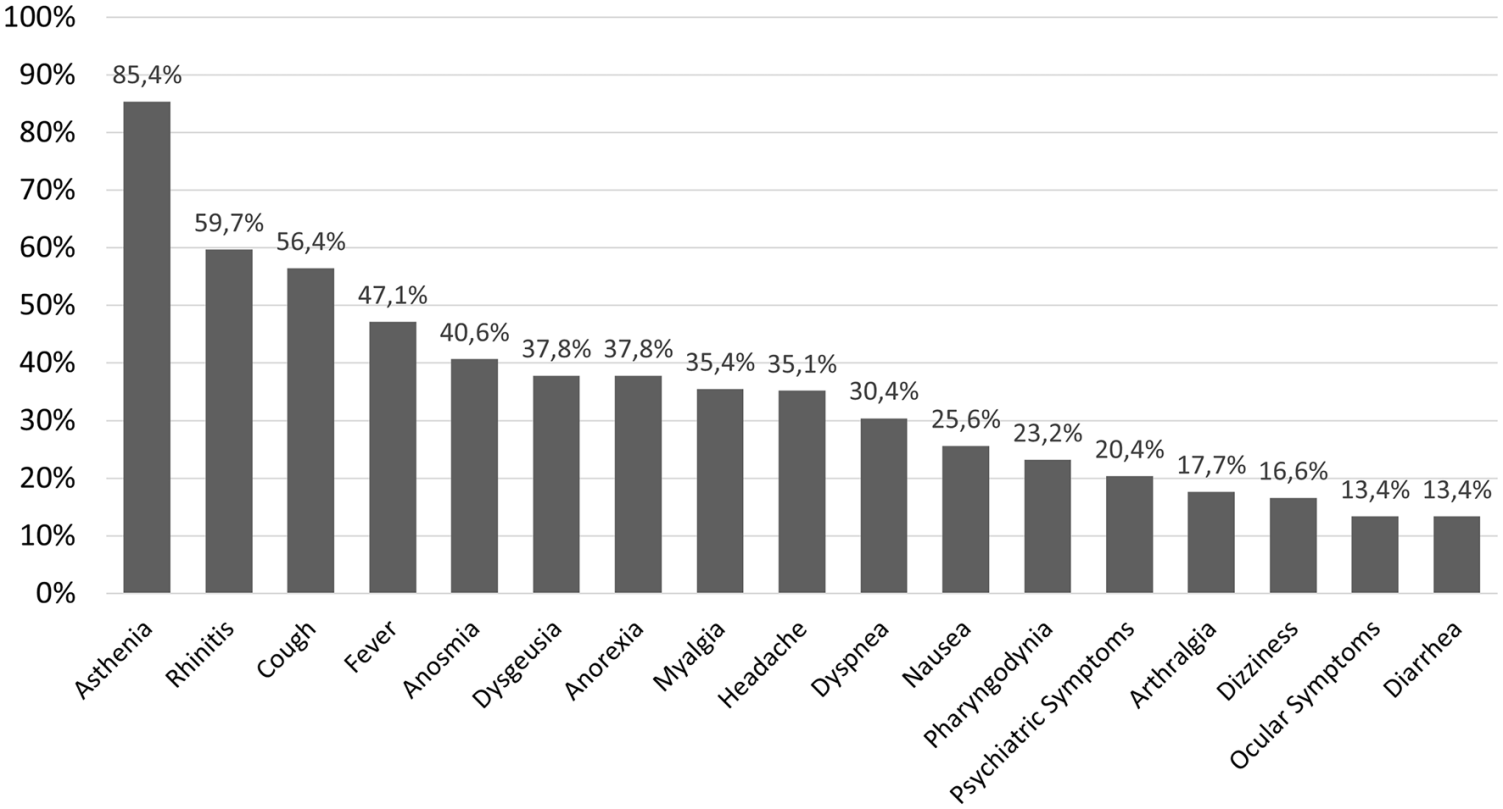
Prevalence of symptoms reported by mild-to-moderate COVID-19 patients

The prevalence of the different symptoms is shown in Fig. 1. The main symptoms reported by the patients were asthenia (*N* = 1291, 85.4%), rhinitis (*N* = 902, 59.7%), and cough (*N* = 853, 56.4%), followed by fever (*N* = 712, 47.1%), anosmia (*N* = 614, 40.6%), dysgeusia (*N* = 571, 37.8%), anorexia (*N* = 571, 37.8%), myalgia (*N* = 536, 35.4%), and headache (*N* = 531, 35.1%).

A total of 251 (16.6%) patients reported dizziness, among whom 110 (43.8%) complained of lightheadedness, 70 (27.9%) complained of disequilibrium, 41 (16.3%) complained of presyncope, and 30 (12%) complained of vertigo.

The frequency of dizziness and of its subcategories in relation to sex, age group, smoking and BMI are reported in Table 2. Compared to males, females had a higher prevalence of vertigo (2.7% vs 1.2%, *P* = 0.03), presyncope (3.7% vs 1.7%, *P* = 0.02) and lightheadedness (9.7% vs 4.8%, *P* < 0.001); disequilibrium was more common in former smokers (*P* < 0.001) and older adults (*P* < 0.001); a higher prevalence of syncope was observed in obese patients (*P* = 0.04); and dizziness was overall more frequent in females (20.3 vs 12.9%, *P* < 0.001) and former smokers *(P* = 0.04)*.*

**Table 2:** Characteristics and frequency of dizziness and its subtypes in mild-to-moderate COVID-19 patients

Variable	Patients	Vertigo	*P* value	Disequilibrium	*P* value	Presyncope	*P* value	Lightheadedness	*P* value	Dizziness (total)	*P* value
	*N*	*N* (%)		*N* (%)		*N* (%)		*N* (%)		*N* (%)	
Sex											
Female	765	21 (2.7)	0.03*	32 (4.2)	0.40	28 (3.7)	0.02*	74 (9.7)	< 0.001*	155 (20.3)	< 0.001*
Male	747	9 (1.2)		38 (5.1)		13 (1.7)		36 (4.8)		96 (12.9)	
Age (years)											
< 40	417	8 (1.9)	0.90	10 (2.4)	< 0.001*	5 (1.2)	0.07	37 (8.9)	0.20	60 (14.4)	0.16
40–59	595	13 (2.2)		21 (3.5)		18 (3.0)		44 (7.4)		96 (16.1)	
≥ 60	500	9 (1.8)		39 (7.8)		18 (3.6)		29 (5.8)		95 (19.0)	
Smoking											
Never	1011	21 (2.1)	0.88	31 (3.1)	< 0.001*	30 (3.0)	0.56	74 (7.3)	0.99	156 (15.4)	0.04*
Former	363	7 (1.9)		34 (9.4)		9 (2.5)		26 (7.2)		76 (20.9)	
Current	138	2 (1.4)		5 (3.6)		2 (1.4)		10 (7.2)		19 (11.1)	
BMI (kg/m^2^)											
< 18.5	27	1 (3.7)	0.32	0 (0.0)	0.60	0 (0.0)	0.04*	2 (7.4)	0.78	3 (8.8)	0.21
18.5–24.9	740	19 (2.6)		32 (4.3)		20 (2.7)		57 (7.7)		128 (17.4)	
25.0–29.9	643	8 (1.2)		33 (5.1)		14 (2.2)		42 (6.5)		97 (15.2)	
** > **30	102	2 (2.0)		5 (4.9)		7 (6.9)		9 (8.8)		23 (22.6)	

*Indicates statistically significant difference, *P* value < 0.05

Lightheadedness was significantly correlated with fever (*P* = 0.003), headache (*P* < 0.001), cough (*P* = 0.02), dyspnea (*P* < 0.001), psychiatric symptoms (*P* < 0.001), pharyngodynia (*P* < 0.001), myalgia (*P* = 0.02), dysgeusia (*P* < 0.001), and anorexia (*P* < 0.001) (Table 3). Table 3 also shows positive correlations between disequilibrium and headache (*P* = 0.008), asthenia (*P* = 0.03), myalgia (*P* < 0.001), arthralgia (*P* < 0.001), anorexia (*P* = 0.008), and ocular symptoms (*P* < 0.001), between presyncope and asthenia (*P* = 0.007), anorexia (*P* < 0.001), diarrhea (*P* < 0.001), and nausea (*P* < 0.001), and between vertigo and headache (*P* = 0.001), anorexia (*P* < 0.001), nausea (*P* < 0.001), and ocular symptoms (*P* < 0.001).

**Table 3:** Prevalence of vertigo, disequilibrium, presyncope and lightheadedness according to selected symptoms in mild-to-moderate COVID-19 patients

Variable	Patients	Vertigo	*P* value	Disequilibrium	*P* value	Presyncope	*P* value	Lightheadedness	*P* value
	*N*	*N* (%)		*N* (%)		*N* (%)		*N* (%)	
Fever									
Yes	712	14 (2.0)	0.96	39 (5.5)	0.14	25 (3.5)	0.07	67 (9.4)	0.003*
No	800	16 (2.0)		31 (3.9)		16 (2.0)		43 (5.4)	
Headache									
Yes	531	19 (3.6)	0.001*	35 (6.6)	0.008*	13 (2.4)	0.64	66 (12.4)	< 0.001*
No	981	11 (1.1)		35 (3.6)		28 (2.9)		44 (4.5)	
Cough									
Yes	853	14 (1.6)	0.28	43 (5.0)	0.39	24 (2.8)	0.78	74 (8.7)	0.02*
No	659	16 (2.4)		27 (4.1)		17 (2.6)		36 (5.5)	
Dyspnea									
Yes	460	6 (1.3)	0.21	24 (5.2)	0.47	9 (2.0)	0.23	50 (10.9)	< 0.001*
No	1052	24 (2.3)		46 (4.4)		32 (3.0)		60 (5.7)	
Psychiatric symptoms									
Yes	308	6 (1.9)	0.96	19 (6.2)	0.15	9 (2.9)	0.80	61 (19.8)	< 0.001*
No	1204	24 (2.0)		51 (4.2)		22 (1.8)		49 (4.1)	
Asthenia									
Yes	1291	27 (2.1)	0.47	66 (5.1)	0.03*	41 (3.2)	0.007*	99 (7.7)	0.16
No	221	3 (1.4)		4 (1.8)		0 (0.0)		11 (5.0)	
Pharyngodynia									
Yes	351	2 (0.6)	0.08	19 (5.4)	0.43	8 (2.3)	0.57	36 (10.3)	< 0.001*
No	1161	28 (2.4)		51 (4.4)		33 (2.8)		74 (6.4)	
Rhinitis									
Yes	902	14 (1.6)	0.14	39 (4.3)	0.49	23 (2.5)	0.64	66 (7.3)	0.94
No	610	16 (2.6)		31 (5.1)		18 (3.0)		44 (7.2)	
Myalgia									
Yes	536	15 (2.8)	0.09	54 (10.1)	< 0.001*	15 (2.8)	0.88	50 (9.3)	0.02*
No	976	15 (1.5)		16 (1.6)		26 (2.7)		60 (6.1)	
Arthralgia									
Yes	267	5 (1.9)	0.89	36 (13.5)	< 0.001*	7 (2.6)	0.92	27 (10.1)	0.05
No	1245	25 (2.0)		34 (2.7)		34 (2.7)		83 (6.7)	
Anosmia									
Yes	614	14 (2.3)	0.50	35 (5.7)	0.10	19 (3.1)	0.45	48 (7.8)	0.50
No	898	16 (1.8)		35 (3.9)		22 (2.4)		62 (6.9)	
Dysgeusia									
Yes	571	13 (2.3)	0.53	32 (5.6)	0.16	17 (3.0)	0.62	59 (10.3)	< 0.001*
No	941	17 (1.8)		38 (4.0)		24 (2.6)		51 (5.4)	
Anorexia									
Yes	571	26 (4.6)	< 0.001*	37 (6.5)	0.008*	30 (5.3)	< 0.001*	64 (11.2)	< 0.001*
No	941	4 (0.4)		33 (3.5)		11 (1.2)		46 (4.9)	
Diarrhea									
Yes	202	3 (1.5)	0.59	11 (5.4)	0.55	16 (7.9)	< 0.001*	21 (10.4)	0.07
No	1310	27 (2.1)		59 (4.5)		25 (1.9)		89 (6.8)	
Nausea									
Yes	387	20 (5.2)	< 0.001*	20 (5.2)	0.56	22 (5.7)	< 0.001*	37 (9.6)	0.05
No	1125	10 (0.9)		50 (4.4)		19 (1.7)		73 (6.5)	
Ocular symptoms									
Yes	203	11 (5.4)	< 0.001*	24 (11.8)	< 0.001*	9 (4.4)	0.11	21 (10.3)	0.07
No	1309	19 (1.5)		46 (3.5)		32 (2.4)		89 (6.8)	

*Indicates statistically significant difference, *P* value < 0.05

## Discussion

The present study investigated in detail the prevalence and pathophysiological mechanisms of the different subtypes of balance disorders in a large sample of COVID-19 patients.

Several symptoms reported by mild-to-moderate COVID-19 patients, such as headache, anorexia, myalgia, arthralgia, asthenia, cough, and fever, are not specific for SARS-CoV-2 and have been described for other infections, especially common flu or influenza [17, 18]. However, the distribution of angiotensin-2 converting enzyme (ACE-2) receptors could explain the peculiar characteristics of SARS-CoV-2 infection, including the different incubation periods, symptom prevalence, and clinical evolution [18].

Compared with previous studies of mild-to moderate COVID-19 patients [3, 19], we found that asthenia was the most commonly reported symptom, followed by rhinitis and cough.

In the present study, dizziness was a relevant symptom that was more common than other symptoms currently considered suggestive of COVID-19, such as diarrhea and conjunctivitis [4, 5]. The prevalence of dizziness (16.6%) was similar to that reported in the preliminary results by other authors [6, 7]; however, the sample of our study was much larger and the investigation was performed through open-ended and targeted questions posed by physicians directly involved in the routine diagnosis of balance disorders. Therefore, the specificity and consistency of the data collected allow us to confirm that dizziness should be included among the main symptoms of COVID-19.

Interestingly, females were more affected than males (20.3% versus 12.9%), and contrary to what might have been expected [20], significant differences in prevalence were not observed among the different age groups.

In clinical practice, careful history records are essential to correctly distinguish vertigo from presyncope, disequilibrium and lightheadedness [12, 14–16]. Telephone interviews have been demonstrated to have good specificity and sensitivity in the diagnosis of vestibular disorders [21]. The prevalence and pathophysiological mechanisms of the different types of dizziness in patients with SARS-CoV-2 infection evaluated in this study are discussed in detail in the following sections.

Lightheadedness was reported by 7.3% of monitored patients and represented the primary cause of dizziness (43.8% of the total cases). Curiously, the prevalence of lightheadedness was much higher in females than in males, which was possibly due to the complex and still underexplored role of the endocrine system in balance function [22].

Psychiatric disorders, such as panic and phobic disorders, generalized anxiety and depression, have been described as common psychological reactions to the COVID-19 pandemic [23] and are considered common causes of nonspecific dizziness [12]. In particular, emotional stress due to SARS-CoV-2 infection and mandatory quarantine might lead to increased serum levels of cortisol and adrenaline, and decreased serotonin, as well as to hyperventilation-induced hypocapnia, which are potentially associated with lightheadedness [24, 25].

Another common cause of lightheadedness is hypoglycemia. Prolonged fasting and anorexia during SARS-CoV-2 infection due to systemic inflammation, dysgeusia, pharyngitis, anxiety and poor motivation might lead to unintentional weight loss, malnutrition, and hypoglycemia, which represent possible trigger mechanisms for the “cytokine storm” [26, 27].

Consequently, the strong positive correlations observed in the present study between lightheadedness and psychiatric symptoms, dyspnea, anorexia, pharyngodynia, dysgeusia and headache are not surprising.

Disequilibrium was the second cause of dizziness (27.9% of total cases) and affected 6.6% of patients with SARS-CoV-2 infection. It was more common in elderly patients and former smokers, thus confirming that aging is an important risk factor for disequilibrium [14, 20].

Balance control is the result of continuous and complex multisensory interactions among visual, somatosensory and vestibular inputs, which are integrated at different levels in the central nervous system, especially at the brainstem and cerebellum [28]. Therefore, injuries, degenerations or transitory functional alterations, even minimal, due to COVID-19 in one (or more) of the three subsystems or in specific areas of the central nervous system can lead to disequilibrium.

Balance and postural stability are typically reduced in patients with visual impairment from underlying eye disease or eye movement disorders [29]. Follicular conjunctivitis, retinal anatomical alterations and peripheral nerve palsies are possible ophthalmological manifestations due to COVID-19 that could prevent the visual system from maintaining the optimal postural balance [30]. Accordingly, we found a strong positive correlation between disequilibrium and ocular symptoms in COVID-19 patients.

Several musculoskeletal manifestations of COVID-19 have also been described in the literature, such as myositis, neuropathy and arthropathy [31, 32]. SARS-CoV-2 has been hypothesized to damage the muscles by directly binding to the ACE-2 receptor or different immune-mediated mechanisms, including release of myotoxic cytokines and deposition of immune complexes [31, 32]. Peripheral neuropathy could be explained by the interaction of SARS-CoV-2 with the ACE-2 receptor, by “molecular mimicry” or by the direct cytotoxicity of the virus on the nerves [31, 32]. Viral arthritis, reactive arthritis or chronic arthritis, such as rheumatoid arthritis and spondyloarthritis, may also be induced or triggered by SARS-CoV-2 infection through a variety of mechanisms, such as cytokine storm, Th17 shift, and immune surveillance escape [33]. These hypotheses might explain the strong positive correlations shown by this study between disequilibrium and arthralgia and myalgia in COVID-19 patients.

The prevalence of presyncope among patients with SARS-CoV-2 was slightly higher than that previously reported for patients after influenza (2.7 vs 2.2%, respectively) [34], with females typically more affected than males [15]. The percentage of dizziness attributable to presyncope was not negligible (16.3%).

Most syncopal episodes in COVID-19 patients have been etiologically classified as unspecified, with no increased risk of adverse outcomes [35, 36]. SARS-CoV-2 infection might be responsible for presyncope/syncope through several mechanisms, including viral myocarditis, adrenergic denervation, autoimmune autonomic neuropathy, and dehydration due to sweating, vomiting, diarrhea, low water intake or medications (such as angiotensin receptor blocking agents) [34–36].

The present study seems to confirm these hypotheses, thus showing strong positive correlations between presyncope and anorexia, diarrhea and nausea.

Vertigo was reported by only 2.0% of monitored COVID-19 patients, accounting for 12% of dizziness cases. As expected [16], females were significantly more affected than males.

Anorexia, nausea and headache were significantly associated with vertigo, which are typical consequences of acute attacks. Based on international criteria, vertigo can be divided into five subgroups: benign paroxysmal positional vertigo (BPPV), vestibular neuritis, labyrinthitis, Menière's disease, and vestibular migraine [16].

*Benign paroxysmal positional vertigo* (BPPV) is considered the most common cause of vertigo [16]. It has been shown that several viruses, such as herpes simplex virus (HSV), cytomegalovirus, Epstein-Barr virus and adenovirus, can not only damage the vestibular nerve and the vestibular membranous labyrinth [37], but also promote BPPV attacks [38]. Likewise, SARS-CoV-2 might cause degeneration of the utricular otolithic membrane and migration of free-floating otoconial debris in the semicircular canals. Lack of physical activity during mandatory quarantine due to SARS-CoV-2 positivity may also be a risk factor for BPPV [39].

*Vestibular neuritis* is an acute peripheral vestibulopathy, possibly caused by viral infection or reactivation of HSV1 in the vestibular ganglia [14]. SARS-CoV-2 infection could affect the vestibular portion of the eighth cranial nerve by direct damage, interaction with ACE-2 receptors, ischemia of the vasa nervorum, inflammatory demyelination, or weakening of the immune defenses that would favor reactivation of latent herpes simplex virus, especially HSV1 [40–42]. When SARS-CoV-2 also affects the cochlear branch of the vestibulocochlear nerve, the infection could result in *labyrinthitis*, which is typically characterized by the combination of vertigo with unilateral hearing loss [43].

The etiopathology of *Menière's disease* is considered multifactorial. However, a possible association with viral infections has been described by some studies [44, 45]. SARS-CoV-2 might play a role in the pathogenesis of Menière's through the elevation of plasma arginine vasopressin (pAVP) caused by stress due to COVID-19 and forced quarantine and by inducing an inflammatory state that could lead to the production of autoantibodies against the endolymphatic sac [45, 46].

The recurrent episodes of migraine, which characterized *vestibular migraine,* could be explained by activation of the trigeminovascular system due to systemic hyperinflammation or endotheliitis following the direct interaction of SARS-CoV-2 and ACE-2 in the meningeal endothelium [47]. Physical and psychological stress, hormonal fluctuations, sensory dysregulation, insomnia and fasting due to COVID-19 may also trigger migraine attacks [48].

### Limitations and future prospects

This study has several limitations. First, it is based on patient reports during the telephone consultation and not on objective clinical examinations. Furthermore, some patients might have omitted or emphasized symptoms, have taken medications without reporting it to physicians, or have had concomitant occult disorders. Another important limitation of the study is the short evaluation period, limited only to days of SARS‐CoV‐2 nasopharyngeal swab positivity, with no subsequent follow-up. Further studies on patients examined directly at the hospital are necessary to confirm our findings. A possible future development of this research could involve the clinical evaluation of the long-term consequences of COVID-19 on the vestibular system.

## Conclusion

The present study specifically assessed the different types of balance disorders in a large sample of COVID-19 patients, and the findings suggest that dizziness should be considered a main symptom that deserves investigation and monitoring during the period of acute SARS-CoV-2 infection. Indeed, one-sixth of the patients in this series complained of new-onset dizziness, with females being significantly more affected than males.

Most cases were attributable to lightheadedness, probably due to psychological and metabolic causes exacerbated by stress following acute infection and mandatory quarantine.

Particular attention should be given to the detection of disequilibrium in the elderly, especially in the presence of myalgia, arthralgia, asthenia and ocular symptoms, due to the possible risk of accidental falls and consequent severe injuries.

Direct, ischemic, hypoxic, and immune-mediated damage are the most likely mechanisms underlying vestibular symptoms, often explained by the interaction between SARS-CoV-2 and ACE-2 receptors.

Awareness of possible balance disorders among COVID-19 patients is of paramount importance for promoting specific diagnostic assessment and rehabilitative procedures to prevent immediate and long-term consequences.

## Data Availability

The source data are available on request.
